# Eye and adnexa hospitalization in Australia: An ecological study

**DOI:** 10.1097/MD.0000000000038829

**Published:** 2024-07-05

**Authors:** Fadi Fouad Hassanin, Abdallah Y. Naser, Waseem A. Aalam, Mehenaz Hanbazazh

**Affiliations:** a Department of Ophthalmology, College of Medicine, University of Jeddah, Jeddah, Saudi Arabia; b Department of Applied Pharmaceutical Sciences and Clinical Pharmacy, Faculty of Pharmacy, Isra University, Amman, Jordan; c Department of Pathology, College of Medicine, University of Jeddah, Jeddah, Saudi Arabia.

**Keywords:** admission, adnexa, Australia, ecological, eye, hospitalization, ocular adnexa

## Abstract

To investigate the trends of hospital admissions concerning diseases of the eye and adnexa in Australia in the past 2 decades. This is a descriptive ecological study on the population level that examined hospitalization data for the duration between 1998 and 2021 in Australia. Hospitalization data were extracted from the National Hospital Morbidity Database. The chi-squared test was utilized to assess the difference in admission rates between the years 1998 and 2021. Hospital admission rate for diseases of the eye and adnexa increased by 1.20-fold (from 852.32 [95% confidence interval [CI] 848.16–856.47] in 1998 to 1873.72 [95% CI 1868.48–1878.96] in 2021 per 100,000 persons, *P* < .01). The most common cause of hospitalization for diseases of the eye and adnexa was disorders of the lens (65.7%), followed by disorders of the choroid and retina (15.6%), followed by disorders of the eyelid, lacrimal system, and orbit (7.7%). Hospital admission rate among males increased by 1.25-fold (from 737.67 [95% CI 732.18–743.16] in 1998 to 1657.19 [95% CI 1650.19–1664.20] in 2021 per 100,000 persons). Hospital admission rate among females increased less sharply by 1.03-fold (from 965.37 [95% CI 959.14–971.59] in 1998 to 1964.35 [95% CI 1956.80–1971.90] in 2021 per 100,000 persons). There are clear gender and age roles in the epidemiology of hospital admissions related to eye and adnexa disorders. Lens disorders were the most common cause of hospital admission. The admission rate increase during the past decades could be due to increases in life expectancy, lifestyle changes, and improvements in screening protocols.

## 1. Introduction

People who live long enough will have at least 1 eye disorder in their lifetime, making eye disorders relatively common.^[1]^ The World Health Organization estimates that there are at least 2.2 billion people worldwide who are blind or have vision impairment, and at least 1 billion of them have diseases that are treatable or preventable.^[1]^ More than 13 million Australians are estimated to suffer from one or more chronic eye conditions.^[2]^

According to a recent study, people over the age of 50 are more likely to have visual impairment.^[3]^ The world population is aging quickly. Similar trends may be seen in Australia, where it is predicted that by 2066, 25% of the population will be 65 years or older.^[4]^ Australians aged 65 and older have higher health care needs than younger Australians, with 50% more hospital days spent there, a 4-fold higher rate of hospital admissions, and a 2-fold higher rate of general practitioner visits.^[5]^

Even though they are almost universally more common in the elderly in Australia, chronic eye problems afflict people of all ages, with just 12% of those under the age of 15 being affected, compared to 93% of those 65 and more in 2017 to 2018.^[2]^ In Australia, persons 75 years of age and older experienced the greatest increase in hospital admission rates for eye illnesses between 2017 and 2019.^[6]^ In England and Wales, the age group of 60 to 74 years old experienced the largest increase in hospital admission rates for eye and adnexa diseases between 1999 and 2019.^[7]^ In Australia, men are less likely than women to suffer chronic eye conditions (51% vs 59%, respectively).^[6]^ In England and Wales, females were more likely than men to be admitted to the hospital overall for eye and adnexa illnesses between 1999 and 2019.^[7]^ Due to urbanization, lifestyle changes, behavioral shifts, population aging, and growth, there will be a significant increase in the number of persons with blindness, vision impairment, and eye problems in the upcoming decades.^[1,8]^ Therefore, the purpose of this study was to examine the pattern of hospital admissions for eye and adnexa diseases in Australia over the last 23 years.

## 2. Materials and methods

### 2.1. Study design

This is a descriptive ecological study on the population level that examined hospitalization data for the duration between 1998 and 2021 in Australia.

### 2.2. Data sources

#### 2.2.1. National hospital morbidity database

The National Hospital Morbidity Database (NHMD) is part of the National Hospitals Data Collection. National Hospitals Data Collection includes the major national hospital databases held by the Australian Institute of Health and Welfare.^[9]^ NHMD is an online database in which data provided by state and territory health authorities in Australia is collected.^[10]^ The data collected at the NHMD are sets of episodes-level records from the morbidity data collection systems of patients admitted to private and public hospitals in Australia. The data provided are contingent on the national minimum data set (NMDS) for admitted patient care and contain data on the following: diagnoses of the patients, external reasons for poisoning and injury, administrative and length of stay, procedures they underwent in the hospital, and demographic. The collection of details about the care provided to hospitalization patients in Australian hospitals is the goal of NMDS for admitted patient care. The coverage of the NMDS is episodes of care for hospital admission patients in all alcohol and drug treatment centers, free-standing day hospital facilities, and private and public psychiatric and acute hospitals.^[11–14]^

#### 2.2.2. Australian bureau of statistics

The Australian Bureau of Statistics are the Australian National Statistics Agency and the official source for reliable and independent information.^[4,15]^ Australian Bureau of Statistics was used to collect mid-year population data for the period between 1998 and 2021 as follows: the historical population was used to collect population data between 1998 and 2016. National, state and territory population were used to collect population data between 2017 and 2021.^[4,15]^

### 2.3. Study population

All private and public diseases of the eye and adnexa admissions data from 1998 to 2021 in Australia were included in the study.

### 2.4. Ethics approval

Hospital admissions and population data are publicly available as de-identified data. Therefore, it was considered an exempt category. All methods were performed in accordance with the relevant guidelines and regulations and there was no need to obtain participants consent as we used publicly available anonymized data on the population level with no personal data shared or analyzed.

### 2.5. Statistical analysis

The estimation of admission rates was conducted by dividing the number of admissions by the mid-year population, and the results were reported alongside 95% confidence intervals (CIs). The estimation of age-specific admission rate was conducted by dividing the total number of admission episodes for each specific age group by the mid-year population of that particular age group in the corresponding year. The gender-specific admission rate was estimated using a consistent technique, which involved dividing the number of admission episodes for each gender (males or females) by the mid-year population of males or females in the corresponding year. The chi-squared test was utilized to assess the difference in admission rates between the years 1998 and 2021. The analyses were performed using SPSS version 27, developed by IBM Corp, located in Armonk, NY, USA.

## 3. Results

### 3.1. Hospital admissions profile during the study period

Between the years 1998 and 2021, Australia documented a cumulative count of 6669,768 instances of admission incidents. The annual number of hospital admissions for various causes experienced a significant increase over the period from 1998 to 2021. Specifically, there was a 2.01-fold increase in the total number of admissions, rising from 160,340 in 1998 to 482,260 in 2021. This increase also resulted in a corresponding rise in the hospital admission rate, which increased by 1.20-fold. In 1998, the admission rate was 852.32 (95% CI 848.16–856.47) per 100,000 persons, while in 2021, it reached 1873.72 (95% CI 1868.48–1878.96) per 100,000 persons. This observed increase in hospital admissions and admission rate was statistically significant (*P* < .01).

The proportion of hospital admissions that occurred on the same day as the patient arrival amounted for 91.4% of the total admissions, while the remaining 8.6% required an overnight stay. The rates of hospital admission on the same day of presentation exhibited a 1.76-fold rise, rising from 628.46 (95% CI 624.89–632.03) per 100,000 individuals in 1998 to 1732.01 (95% CI 1726.97–1737.05) per 100,000 individuals in 2021. The data reveals a significant decline in the rates of hospital admissions for overnight stays over the course of 23 years. Specifically, there was a 64.1% decrease, with the rate dropping from 223.86 (95% CI 221.72–225.99) per 100,000 persons in 1998 to 80.34 (95% CI 79.24–81.43) per 100,000 persons in 2021, Figure 1.

**Figure 1.**
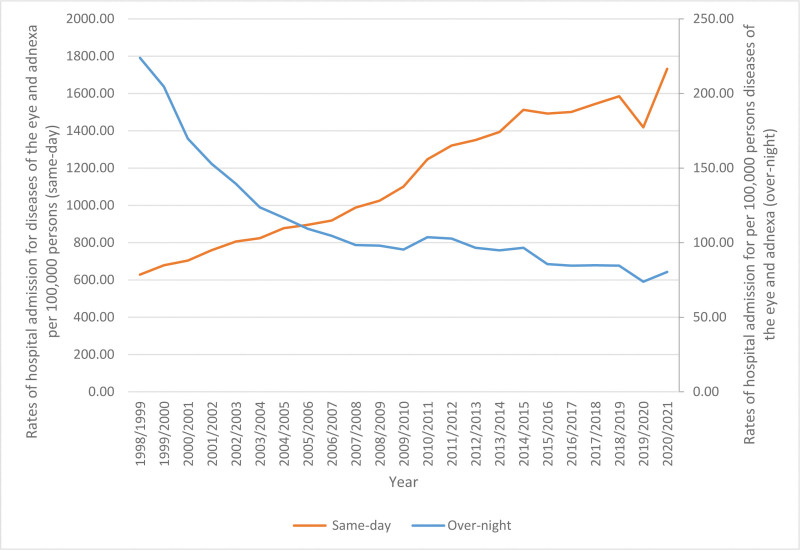
Rates of same-day and overnight-stay patients hospital admission.

The most prevalent cause for hospital admissions was “disorders of lens,” which accounted for 65.7% of the total number of admissions. This was followed by “disorders of choroid and retina” at 15.6%, and “disorders of eyelid, lacrimal system and orbit” at 7.7% (Table 1).

**Table 1:** Percentage of diseases from total number of admissions.

Code	Description	Percentage from total number of admissions
H00–H06	“Disorders of eyelid, lacrimal system and orbit”	7.7%
H10–H13	“Disorders of conjunctiva”	2.9%
H15–H22	“Disorders of sclera, cornea, iris and ciliary body”	1.4%
H25–H28	“Disorders of lens”	65.7%
H30–H36	“Disorders of choroid and retina”	15.6%
H40–H42	Glaucoma	1.8%
H43–H45	“Disorders of vitreous body and globe”	0.9%
H46–H48	“Disorders of optic nerve and visual pathways”	0.3%
H49–H52	“Disorders of ocular muscles, binocular movement, accommodation and refraction”	2.5%
H53–H54	“Visual disturbances and blindness”	0.6%
H55–H59	“Other disorders of eye and adnexa”	0.6%

### 3.2. Hospital admissions rates stratified by indication

**Figure 2.**
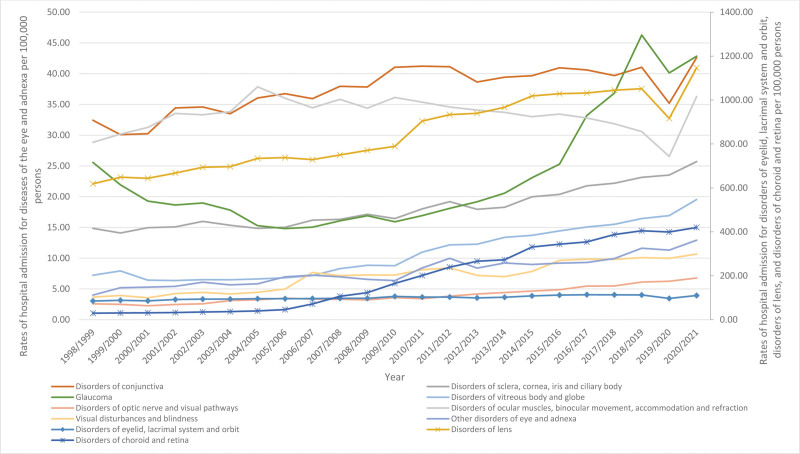
Trends of hospital admission for diseases of the eye and adnexa.

Over the course of the last 23 years, there has been a significant rise in the rate of hospital admissions for various eye-related conditions. Specifically, there has been a notable increase in admissions for disorders of the choroid and retina, other disorders of the eye and its surrounding structures, visual disturbances and blindness, disorders of the vitreous body and globe, as well as disorders of the optic nerve and visual pathways. These conditions have experienced a respective increase of 13.19-fold, 2.23-fold, 1.91-fold, 1.70-fold, and 1.64-fold. Furthermore, the rates of hospital admissions for conditions such as “disorders of lens,” “disorders of sclera, cornea, iris and ciliary body,” glaucoma, “disorders of conjunctiva,” “disorders of eyelid, lacrimal system and orbit,” and “disorders of ocular muscles, binocular movement, accommodation and refraction” exhibited significant increases of 85.3%, 73.1%, 67.5%, 31.4%, 29.5%, and 25.8%, respectively, as indicated in Table 2 and Figure 2.

**Table 2:** Percentage change in the hospital admission rates.

Diseases	Rate of diseases in 1998 per 100,000 persons (95% CI)	Rate of diseases in 2021 per 100,000 persons (95% CI)	Percentage change from 1998 to 2021
“Disorders of eyelid, lacrimal system and orbit”	84.96 (83.64–86.27)	110.04 (108.75–111.32)	29.5%
“Disorders of conjunctiva”	32.43 (31.61–33.24)	42.61 (41.82–43.41)	31.4%
“Disorders of sclera, cornea, iris and ciliary body”	14.85 (14.30–15.40)	25.71 (25.09–26.33)	73.1%
“Disorders of lens”	618.62 (615.07–622.16)	1146.56 (1142.45–1150.68)	85.3%
“Disorders of choroid and retina”	29.58 (28.80–30.36)	419.77 (417.27–422.27)	1319.0%
Glaucoma	25.57 (24.85–26.30)	42.84 (42.04–43.64)	67.5%
“Disorders of vitreous body and globe”	7.22 (6.84–7.61)	19.54 (19.00–20.08)	170.5%
“Disorders of optic nerve and visual pathways”	2.57 (2.34–2.80)	6.78 (6.46–7.10)	163.5%
“Disorders of ocular muscles, binocular movement, accommodation and refraction”	28.85 (28.08–29.62)	36.28 (35.54–37.01)	25.8%
“Visual disturbances and blindness”	3.67 (3.39–3.94)	10.67 (10.27–11.06)	190.8%
“Other disorders of eye and adnexa”	4.00 (3.71–4.28)	12.92 (12.48–13.36)	223.3%

CI = confidence interval.

### 3.3. Hospital admissions stratified by gender

**Figure 3.**
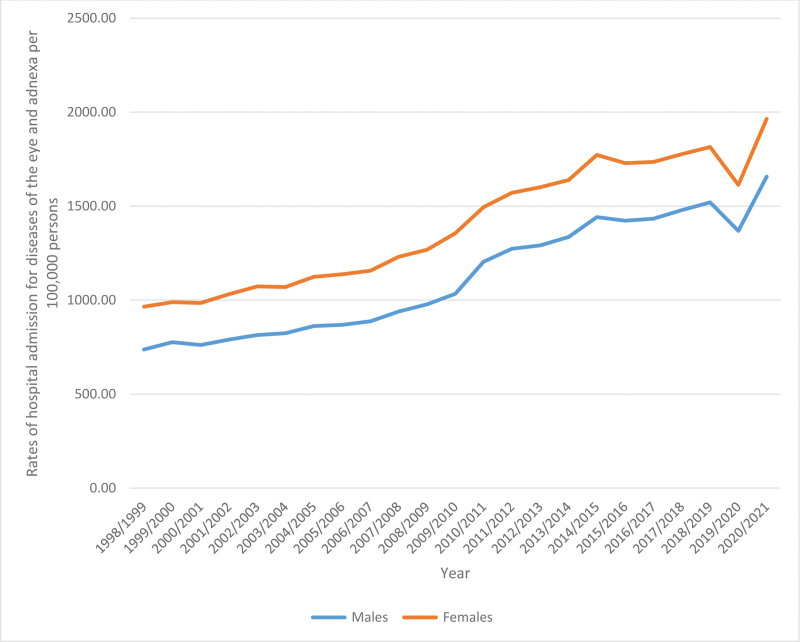
Rates of hospital admission stratified by gender.

Females accounted for 55.8% of the total number of hospital admissions, which amounted to 3654,790 episodes. On average, there were 158,903 episodes per year. The admission rate among males increased by a factor of 1.25, going from 737.67 (95% CI 732.18–743.16) in 1998 to 1657.19 (95% CI 1650.19–1664.20) in 2021 per 100,000 persons. Similarly, the admission rate among females increased by a factor of 1.03, rising from 965.37 (95% CI 959.14–971.59) in 1998 to 1964.35 (95% CI 1956.80–1971.90) in 2021 per 100,000 persons (Fig. 3).

### 3.4. Hospital admissions stratified by age

**Figure 4.**
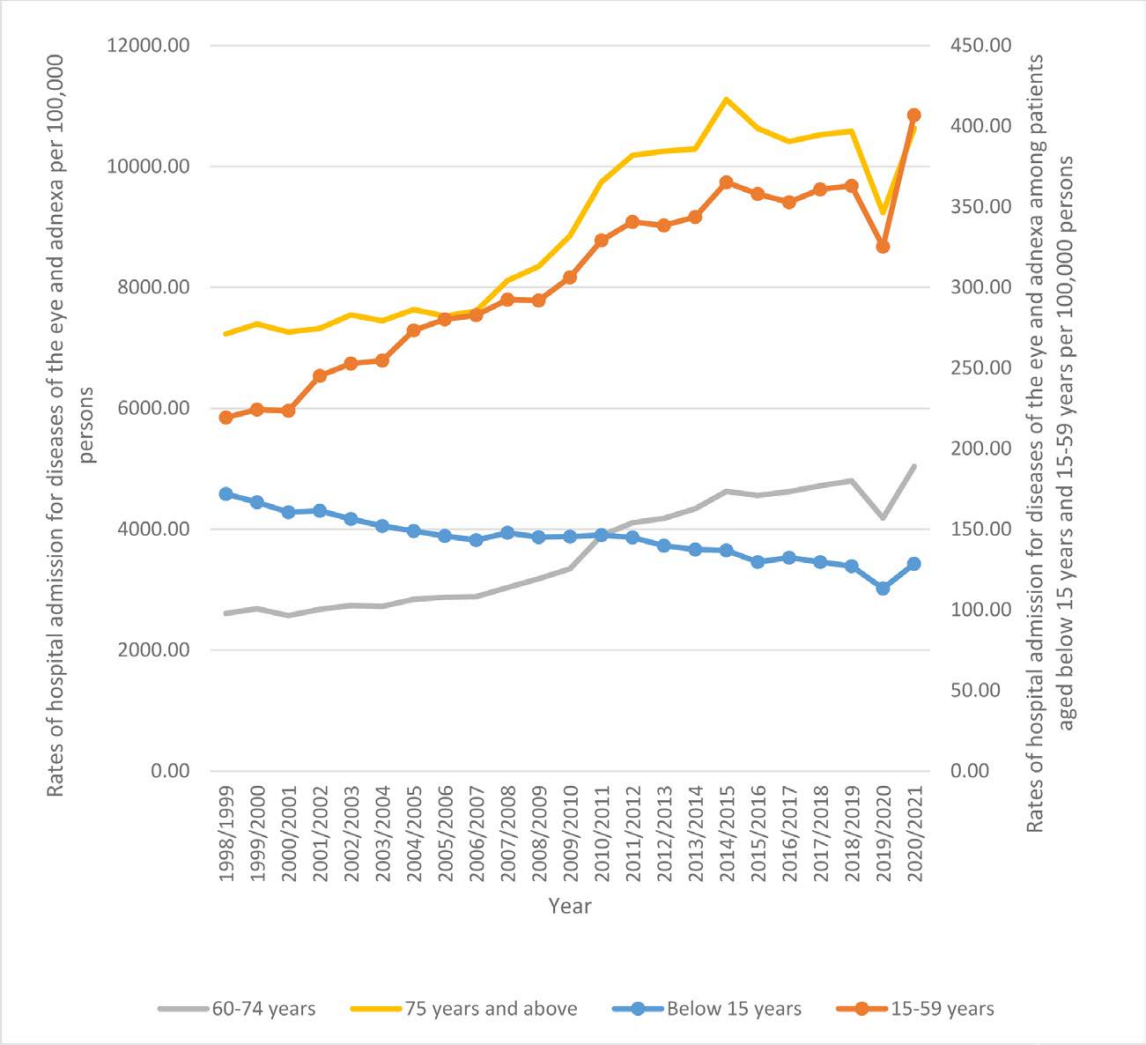
Rates of hospital admission stratified by age group.

In relation to the hospital admissions for diseases of the eye and adnexa, there was a notable distribution of age groups. Specifically, individuals aged 75 years and above constituted the largest proportion, accounting for 45.2% of the total admissions. Following this, the age group of 60 to 74 years represented 37.9% of the admissions, while individuals aged 15 to 59 years comprised 14.8% of the admissions. Lastly, the age group below 15 years constituted a smaller proportion, with only 2.1% of the admissions. The hospital admission rates for individuals under the age of 15 experienced a decline of 25.2% during the course of the study period. Specifically, the rates decreased from 171.86 (95% CI 167.76–175.95) per 100,000 persons in 1998 to 128.53 (95% CI 125.31–131.74) per 100,000 persons in 2021. The hospital admission rates for individuals aged 15 to 59 years experienced a significant increase of 85.6% over the course of 23 years, from 219.26 (95% CI 216.59–221.93) per 100,000 persons in 1998 to 406.89 (95% CI 403.68–410.09) per 100,000 persons in 2021. The hospital admission rates for individuals between the ages of 60 and 74 experienced a significant increase of 93.1% over the course of 23 years, from 2609.11 (95% CI 2587.37–2630.86) per 100,000 persons in 1998 to 5038.12 (95% CI 5016.34–5059.89) per 100,000 persons in 2021. The hospital admission rates for those aged 75 years and older exhibited a significant rise of 47.0% over the course of 23 years, from 7229.27 (95% CI 7179.05–7279.49) per 100,000 persons in 1998 to 10,629.44 (95% CI 10,585.76–10,673.12) per 100,000 persons in 2021, as seen in Figure 4.

## 4. Discussion

To the best of our knowledge, this is the only study to investigate the rate of hospital admission related to the eye and adnexa in Australia in the last 2 decades from 1998 to 2021. This duration also includes the COVID-19 pandemic and lockdown period which affected the general hospital admission rates in Australia and worldwide.

Our study found a 1.20-fold increase in the rate of hospital admission for diseases of the eye and adnexa (from 852.32 in 1998 to 1873.72 in 2021, *P* < .01). This increase is attributed to the rise in Australia population over the years. The population has increased from 18 million in 1997 to more than 25 million currently. This makes it one of the highest populations in the world, in which it is ranked the 55th in regards to population, and the 99th in regards to the rate of population growth.^[16,17]^ There are many reasons attributed to the increase in the Australian population, including the rise in rates of birth (over 90,000 births in Australia in 2021), life expectancy, migration (over 7.6 million migrants in 2020), health awareness, screening programs, and advanced diagnostic strategies.^[17,18]^

The hospitals’ admission in Australia between 2014 to 2015 and 2018 to 2019 had more than a 3% average increase per year. By 2019, there were more than 11 million hospital admissions. However, due to the COVID-19 pandemic, there was a 2.5% decrease in hospital admission in 2019. This was followed by a 6% increase to reach 11.8 million hospitalizations in 2020 to 2021 after the pandemic. This noticeable increase was mainly due to the easing of restrictions on hospitals, especially in regard to elective surgeries which were delayed or postponed during the pandemic.^[19]^ Our study showed that the majority of the total number of eye and adnexa hospital admissions (91.4%) was for same-day disease hospital admission, while only 8.6% was for overnight-stay patients. This increase in same-day hospital admission and decrease in overnight-stay hospital admission could be due to the effect of COVID-19 precautions and minimizing the hospitalization hours to reduce the risk of infection.^[20,21]^ Other possible reasons for this shift from overnight-stay hospital admission to same-day hospital admission could be the progress in the field of medicine has resulted in the emergence of minimally invasive procedures and enhanced surgical techniques. The cost of hospital admissions on the same day is frequently lower for both patients and healthcare systems as compared to overnight stays. If it is medically viable, a significant number of patients express a preference for receiving therapy on an outpatient basis. The inclination toward shorter durations of hospitalization, in conjunction with advancements in healthcare provision, has played a role in the rise of same-day admissions. Moreover, in certain circumstances, the reduction of hospitalization duration can contribute to the mitigation of healthcare-associated infections. Reducing the duration of hospital stays can effectively mitigate the risk of patients being exposed to hospital-acquired infections.

According to the Australian Institute of Health and Welfare analysis in 2017 to 2018, 4% of total hospital admission was related to eye and ocular adnexa. Private hospitals accounted for 70% of eye and adnexal tissue hospitalization, especially for common diseases including cataracts and retinal lesions. In contrast, public hospitals were involved more in treating unusual and emergency eye and adnexal cases (93%).^[2]^

In our study, disorders of lens were the most common diseases of the eye and adnexa hospital admissions reason accounting for 65.7% of the total number of diseases of the eye and adnexa hospital admissions, followed by disorders of choroid and retina with 15.6%, and disorders of eyelid, lacrimal system and orbit with 7.7%. Cataract is one of the most common lens disorders and among the major causes of blindness and visual impairment in Australia along with age-related macular degeneration (AMD), diabetic retinopathy (DR), glaucoma, and uncorrected refractive error. The number of Australians with cataracts has been increasing over the years with aging due to increasing life expectancy and systemic conditions such as diabetes, hypertension, and smoking. In 2010 to 2011, 604,000 Australians (3.5%) had cataracts, while the number increased in 2016 to 2017 to reach 743,000 (3.9%). This makes cataract surgery one of the most common surgeries in Australia and worldwide.^[22,23]^ A Clark et al, evaluated cataract surgeries and their major complications in the Western Australian population over a period of 22 years (between 1980 and 2001). They found that retinal detachment (RD) was the most common complication (0.70 surgery was 0.7%). However, the rate of complications lessened significantly over time mainly due to the changes in operative techniques.^[24]^ RD is a frequent sight-threatening ophthalmic condition implicated with multiple risk factors including age, male sex, and operations.^[25]^ The incidence of RD in Western Australia between 2000 and 2013 ranged from 12.78 to 16.20 cases per 100,000. The majority of the cases contributed to males (64%) and those aged above 40 years (86%) peaking in those aged 60 to 79 years.^[26]^ In the island state of Tasmania, the incidence of RD between 2005 and 2010 was 9.7 per 100,000. The peak decade was 60 to 69 years old, with a higher rate for men. The study also concluded that pseudophakic patients with complicated cataract surgeries are more likely to develop RD.^[27]^

AMD is the most common cause of blindness in elderly people in Australia and other western countries. It impacts life quality and is considered a major cause of financial burden.^[28]^ In 2004, more than 600,000 Australians (age 55 or more) have early or late AMD.^[22]^ With the increase in population age and life expectancy, the rate of AMD is expected to increase. Therefore, improvement of low-vision devices and access to vision rehabilitation centers are essential to cope with this burden.

DR is one of the major causes of blindness worldwide. In 2003, more than 15% of Australian diabetic patients had retinopathy.^[28]^ Almost 60% to 100% of patients with type 2 DM and type 1 DM, respectively, will have diabetic eye disease within the first 2 decades of diagnosis.^[29]^ The prevalence of DR is higher in the older age group and the rate is expected to increase with obesity, western diet, and smoking. Based on the Melbourne Vision Impairment Project (1992–1996) and The Blue Mountains Eye Study (1992–1994), 1 in 3 Australians with diabetes (aged 40 years or more) had DR.^[30,31]^

Age and sex are important factors that contributed to the increased rate of hospital admission due to diseases of the eye and adnexa. Concerning age, we found that the age group 75 years and above accounted for 45.2% of the total number of diseases of the eye and adnexa hospital admissions, followed by the age group 60 to 74 years with 37.9%. This is recognizable as the number of old-aged Australians is growing and it represents a large portion of the total population. In 1970, older Australians (aged 65 or more) comprised around 8.3% of the total population. The percentage increased in 1995 and 2020 and reached 12% and 16%, respectively. This provides insights into the high need for old-age services and amenities in the future. By the end of June 2020, more than 50% of the older Australians were aged 65% to 74%, and over half of them were females (88.1 males for every 100 females).^[32]^ This high ratio of females to males is evident in our study, as females contributed to 55.8% of the total number of diseases of the eye and adnexa hospital admission. In 2021, there are 12.84 million (49.79%) and 12.94 million (50.21%) Australian males and females, respectively.^[17]^ It is recognizable across the world and in several species that females live longer than males and this is apparent in the higher percentage of older females and accordingly, higher older male mortality rate. Several factors contribute to this difference in life span including hormonal (the protective effect of estrogen), genetics (two X chromosomes), immunological, cardiovascular, and behavioral.^[33–35]^ Hormonal changes (estrogen in females and androgen in males) affect the prevalence of eye and ocular disorders. This is due to the presence of sex hormone receptors in the ocular tissue. For example, the estrogen fluctuation in females during menstruation, pregnancy, and menopause influence the status of uveitis. Additionally, estrogen has a protective effect against glaucoma complications. However, it is found to progress DR in females.^[7,36,37]^

On the other hand, we found that diseases of the eye and adnexa hospital admission rate among males increased by 1.25-fold (from 737.67 in 1998 to 1657.19 in 2021), while increased by 1.03-fold among females (from 965.37 in 1998 to 1964.35 in 2021). This might be related to the behavioral effect in males as males have a higher rate of ocular trauma and accidents being more adventurous and have unsafe jobs. Multiple studies have found higher rate of hospitalization among male due to eye injuries.^[38–42]^ In 2017 to 2018, males had a higher rate of ocular injury-related hospital admission (58%) than females.^[2,35]^ As a result, identifying risk factors and promoting preventive methods are essential to avoid ocular trauma and insure eye safety.

To the best of our knowledge, this is the first large population study in Australia to provide detailed hospital admission rate for all diseases of the eye and adnexa for a period of 20 years. This will help in monitoring eye disorders in Australia and hence improve the eye health care system. This study has limitations. Given the inherent characteristics of this study design, which is ecological study on the population level, we encounter challenges in effectively managing the variables, exposure factor, and potential confounders, including other comorbidities. The estimations of hospital admission rates in our study encompassed both initial admissions and subsequent readmissions, perhaps resulting in an overestimation of these rates. In addition, the denominator utilized in the calculation of admission rates was the total population size. Therefore, our findings should be interpreted carefully.

Persistent advocacy for early intervention and periodic eye exams for diseases like DR and glaucoma will help decrease the number of eye and adnexa hospitalizations. Complications may also be prevented by encouraging the use of protective eyewear. Patient education programs on ocular health should also be constructed. Combining telemedicine for follow-ups and enhancing the collaboration between primary care and ophthalmology would ensure the delivery of adequate and timely treatment, shifting the need for hospitalization.

## 5. Conclusion

Hospital admissions related to eye and adnexa disorders are common in Australia. There are clear gender and age roles in the epidemiology of hospital admissions related to eye and adnexa disorders. Lens disorders were the most common cause of hospital admission. The admission rate increase during the past decades could be due to increases in life expectancy, lifestyle changes, and improvements in screening protocols.

## Author contributions

**Conceptualization:** Fadi Fouad Hassanin, Abdallah Y. Naser.

**Data curation:** Abdallah Y. Naser.

**Formal analysis:** Abdallah Y. Naser.

**Investigation:** Fadi Fouad Hassanin, Abdallah Y. Naser, Waseem A. Aalam, Mehenaz Hanbazazh.

**Methodology:** Fadi Fouad Hassanin, Abdallah Y. Naser.

**Project administration:** Fadi Fouad Hassanin, Abdallah Y. Naser.

**Resources:** Fadi Fouad Hassanin, Abdallah Y. Naser, Waseem A. Aalam, Mehenaz Hanbazazh.

**Software:** Abdallah Y. Naser.

**Supervision:** Fadi Fouad Hassanin, Abdallah Y. Naser.

**Validation:** Fadi Fouad Hassanin, Abdallah Y. Naser, Waseem A. Aalam, Mehenaz Hanbazazh.

**Visualization:** Fadi Fouad Hassanin, Abdallah Y. Naser, Waseem A. Aalam, Mehenaz Hanbazazh.

**Writing – original draft:** Fadi Fouad Hassanin, Abdallah Y. Naser, Waseem A. Aalam, Mehenaz Hanbazazh.

**Writing – review & editing:** Fadi Fouad Hassanin, Abdallah Y. Naser, Waseem A. Aalam, Mehenaz Hanbazazh.
